# Unexpected no significant soil carbon losses in the Tibetan grasslands due to rodent bioturbation

**DOI:** 10.1093/pnasnexus/pgac314

**Published:** 2022-12-30

**Authors:** Miao Huang, Dezhao Gan, Zheng Li, Jinsong Wang, Shuli Niu, Hongchao Zuo, Ruijun Long, Lei Ma

**Affiliations:** College of Atmospheric Sciences, Lanzhou University, No. 222 Tian-shui South Road, Cheng-guan District, Lanzhou 730000, P.R. China; College of Atmospheric Sciences, Lanzhou University, No. 222 Tian-shui South Road, Cheng-guan District, Lanzhou 730000, P.R. China; College of Ecology, Lanzhou University, No. 222 Tian-shui South Road, Cheng-guan District, Lanzhou 730000, P.R. China; Key Laboratory of Ecosystem Network Observation and Simulation, Institute of Geographical Sciences and Natural Resources Research, Chinese Academy of Sciences, Beijing 100101, P.R. China; Key Laboratory of Ecosystem Network Observation and Simulation, Institute of Geographical Sciences and Natural Resources Research, Chinese Academy of Sciences, Beijing 100101, P.R. China; School of Resources and Environment, University of Chinese Academy of Sciences, Beijing 100049, P.R. China; College of Atmospheric Sciences, Lanzhou University, No. 222 Tian-shui South Road, Cheng-guan District, Lanzhou 730000, P.R. China; College of Ecology, Lanzhou University, No. 222 Tian-shui South Road, Cheng-guan District, Lanzhou 730000, P.R. China; College of Atmospheric Sciences, Lanzhou University, No. 222 Tian-shui South Road, Cheng-guan District, Lanzhou 730000, P.R. China

**Keywords:** rodent bioturbation, soil depth dependence, soil organic carbon, Tibetan grasslands

## Abstract

The Tibetan grasslands store 2.5% of the Earth’s soil organic carbon. Unsound management practices and climate change have resulted in widespread grassland degradation, providing open habitats for rodent activities. Rodent bioturbation loosens topsoil, reduces productivity, changes soil nutrient conditions, and consequently influences the soil organic carbon stocks of the Tibetan grasslands. However, these effects have not been quantified. Here, using meta-analysis and upscaling approaches, we found that rodent bioturbation impacts on the Tibetan grassland soil organic carbon contents were depth-dependent, with significant (*P* < 0.001) decreasing of 24.4% in the topsoil (0 to 10 cm) but significant (*P* < 0.05) increasing of 35.9% in the deeper soil layer (40 to 50 cm), and nonsignificant changes in other soil layers. The depth-dependent responses in soil organic carbon content were closely associated with rodent tunnel burrowing, foraging, excrement deposition, and mixing of the upper and deeper soil layers. Rodent bioturbation had shown nonsignificant impacts on soil bulk density, independent of soil layer. Tibetan grasslands totally lose −35.2 Tg C yr^–1^ (95% CI: −48.5 to −21.1 Tg C yr^–1^) and –32.9 Tg C yr^–1^ (−54.2 to −8.6 Tg C yr^–1^) due to rodent bioturbation in the 0 to 10 or 0 to 30 cm soil layer, while no significant net loss was found over the 0 to 90 cm layer. Our findings highlight the importance of considering depth-dependent factors to robustly quantify the net changes in the terrestrial soil organic carbon stocks resulting from disturbances such as rodent bioturbation.

**Significance Statement**

Rodent bioturbation widely occurs over global grasslands. Tibetan alpine grasslands store 2.5% of the global soil organic carbon (SOC). Minor changes in this pool due to rodent bioturbation may significantly influence atmospheric carbon dioxide concentrations and thus climate warming. Using meta-analysis and upscaling approaches, this study, among the first, shows that the effect of rodent bioturbation on the Tibetan grassland SOC contents and stocks are depth-dependent irrespective of grassland types and rodent species, with significant decreases in topsoil (0 to 10 cm) but increases in the deeper soil layer (40 to 50 cm). This study highlights the importance of considering soil depths in quantifying the grassland SOC stocks changes under rodent bioturbation or anthropogenic activities.

## Introduction

The Tibetan Plateau, which has an average altitude >4,000 m, is the largest plateau in the world and has been called the “Third Pole of Earth” and the “Asian Water Tower” (1). The Tibetan Plateau is comprised primarily of alpine grasslands such as meadows, steppes, and swamp meadows, which together account for approximately 60% of the total area of the plateau (2). Tibetan grasslands is one of the representative grassland ecosystems across Eurasian grasslands (3).These alpine grasslands play crucial roles in the Asian monsoon climate (4), in biodiversity conservation, in the hydrological cycle, and in carbon (C) sequestration (5), as well as in local livestock husbandry (6). The Tibetan grasslands function as significant net C sinks (7, 8) because the cold environment depresses the decomposition of litter and soil organic matter; as a result, large amounts of organic matter are stored in the soils (2, 9). The Tibetan grasslands store 33.52 Pg C (1 Pg = 10^15^ g) soil organic carbon (SOC), accounting for 23.4% of the Chinese SOC stocks and 2.5% of the global SOC stocks (10, 11), but they cover only 15 and 0.3% of the Chinese and total terrestrial area on Earth, respectively (10). Therefore, minor changes in the SOC stocks of the Tibetan grasslands due to anthropogenic disturbances or climate change may result in nonnegligible increases in atmospheric CO_2_ concentrations and thus global warming (12, 13).

The Tibetan grasslands are predominantly used as grazing pastures (14, 15). However, overgrazing has resulted in the widespread degradation of Tibetan grasslands, and approximately 30% of the Tibetan grasslands have been degraded (16). Furthermore, the degradation of these grasslands has been exacerbated by the frequent activity of subterranean rodents such as Plateau zokor (*Myospalax baileyi*) and Plateau pika (*Ochotona curzoniae*). Rodent bioturbation (e.g., tunnel burrowing, foraging for grasses and seeds, and excrement deposition) (17, 18) transfers substantial amounts of belowground soils to the ground surface (19, 20), largely reduces vegetation coverage (21, 22), greatly decreases the net primary productivity of the Tibetan grasslands (23, 24), and changes soil microclimates and nutrient conditions (25, 26). Rodent bioturbation is therefore considered a vital process that impacts the soil carbon cycle (27). Although rodents contribute substantially to soil bioturbation and are globally distributed (28), our understanding of the biogeochemical effects of rodent bioturbation is still insufficient (27, 29). Investigations have shown that rodent bioturbation occurs widely across the Tibetan grasslands, and approximately 150,000 km^2^ (i.e., approximately 10%) of the Tibetan grasslands have been affected by rodent bioturbation (19, 30). We can therefore postulate that rodent bioturbation significantly influences the SOC stocks of the Tibetan grasslands, and further influences global climate change.

Over the past two decades, results from a large number of site-scale measurements have shown that rodent bioturbation significantly decreases SOC contents and stocks in the Tibetan grasslands due to increased soil aeration (31–33). However, most measurements have focused primarily on the topsoil (i.e., 0 to 10 cm), and the rodent bioturbation effects on SOC contents and stocks in the deep soil layers and across entire soil profiles remain largely unknown. Topsoil quantification alone may induce overestimation of the net losses in SOC, as evidence has shown that rodent burrowing activities effectively mix the soils of deeper with upper layers (31, 34); additionally, rodents forage and store grasses and seeds in their nests, and they deposit feces and urine, which returns additional organic C to the deeper soil layers (18, 35). The magnitudes and patterns for changes in SOC content due to rodent bioturbation may differ between grassland types (meadow *vs*. steppe *vs*. swamp meadow) and rodent species (Plateau zokor *vs*. Plateau pika) and may vary from the eastern to western Tibetan Plateau with different climate conditions. However, to date, the magnitudes, patterns, and environmental factors that drive changes in SOC contents due to rodent bioturbation across the Tibetan grasslands have remained unclear. Clarifying the mechanism underlying these changes could contribute to an in-depth understanding of the Tibetan grassland SOC dynamics in response to rodent bioturbation.

In this study, we used meta-analysis and upscaling approaches to synthesize the most extensive paired-measurements (i.e., nonbioturbated grassland site *vs*. neighboring rodent bioturbated site) of SOC contents and soil bulk density (BD) (322 and 132 paired SOC content and BD in-situ observations from 89 studies) across soil profiles (0 to 10, 10 to 20, 20 to 30, 30 to 40, 40 to 50, and 50 to 90 cm) in the Tibetan grasslands to evaluate the rodent bioturbation-driven responses and controlling factors for grassland SOC contents and stocks to rodent bioturbation, and in particular, to understand whether these responses are soil depth-dependent. On this basis, by evaluating rodent bioturbated grassland areas (separated as alpine meadow and alpine steppe) and average bioturbated years, as well as by estimating the net changes (△) in soil organic C density (SOCD) due to rodent bioturbation (i.e., △SOCD = SOCD_bioturbated_–SOCD_nonbioturbated_), we aimed to quantify the net changes in SOC stocks due to rodent bioturbation in the overall soil profile. We hypothesized that rodent bioturbation significantly decreases SOC contents and stocks in topsoil, while significantly increases SOC contents and stocks in the deeper soil layers of the Tibetan grasslands, as the topsoil organic C is likely to be largely decomposed due to rodent bioturbation. Moreover, rodent burrowing and foraging activities mix surface and deeper soil layers and increase SOC contents in the deeper layers. We also hypothesized that rodent bioturbation insignificantly affects the total SOC stocks and tested this hypothesis by considering the net changes in SOC stocks for overall soil profile, as the net losses in SOC in the topsoil could compensate for the net increases in the SOC of the deeper layers. On a global basis, such studies are limited for grassland ecosystems, and this work can provide new insights into grassland SOC dynamics in response to disturbances such as rodent bioturbation.

## Materials and Methods

### Systematic literature review and data compilation

Relevant publications on the effects of rodent bioturbation on soil organic C in the Tibetan grasslands were identified by searching the Web of Science (all databases), Google Scholar, and China National Knowledge Infrastructure Database. The publication years analyzed spanned the period from January 1989 to December 2021. The following combinations of keywords were used for our search: (Qinghai–Tibetan Plateau OR Tibet* Plateau OR Tibet*) with (Rodent* OR Plateau zokor* OR Plateau pika*) with (Soil organic C OR Soil organic matter OR SOC OR SOM OR soil Carbon). Using these keywords, we identified 1,004 relevant publications, which we then screened for relevance and links to other, previously unidentified publications. The following criteria were adopted for considering the addition of studies to our final database:

- The studies needed to compare undisturbed controls with adjacent grasslands that were bioturbated by rodents;
- The studies mainly focused on rodent bioturbation in the Tibetan grasslands, as studies in other countries or other regions of China were scarce;
- Results from in-situ studies were included, while those from soil core or mesocosm incubations were excluded in our meta-analysis, as the former could represent the true impacts of rodent bioturbation;
- The studies provided the mean, SD, or SE, and sample sizes for both the control and treatments;
- Each individual study provided the targeted variable SOC.

After the application of the criteria above, our database ultimately included 89 in-situ studies.

We subsequently extracted the mean data ($\bar{X}$), SD and number of replicates (*n*) from all the studies. If the studies reported SE rather than SD, the SD was calculated by $\;\mathrm{SE}\;\sqrt{n}$. The data were either obtained directly from the tables and texts or extracted by digitizing graphs using Getdata Graph Digitizer software (version 2.26, Russia).

**Fig. 1.**
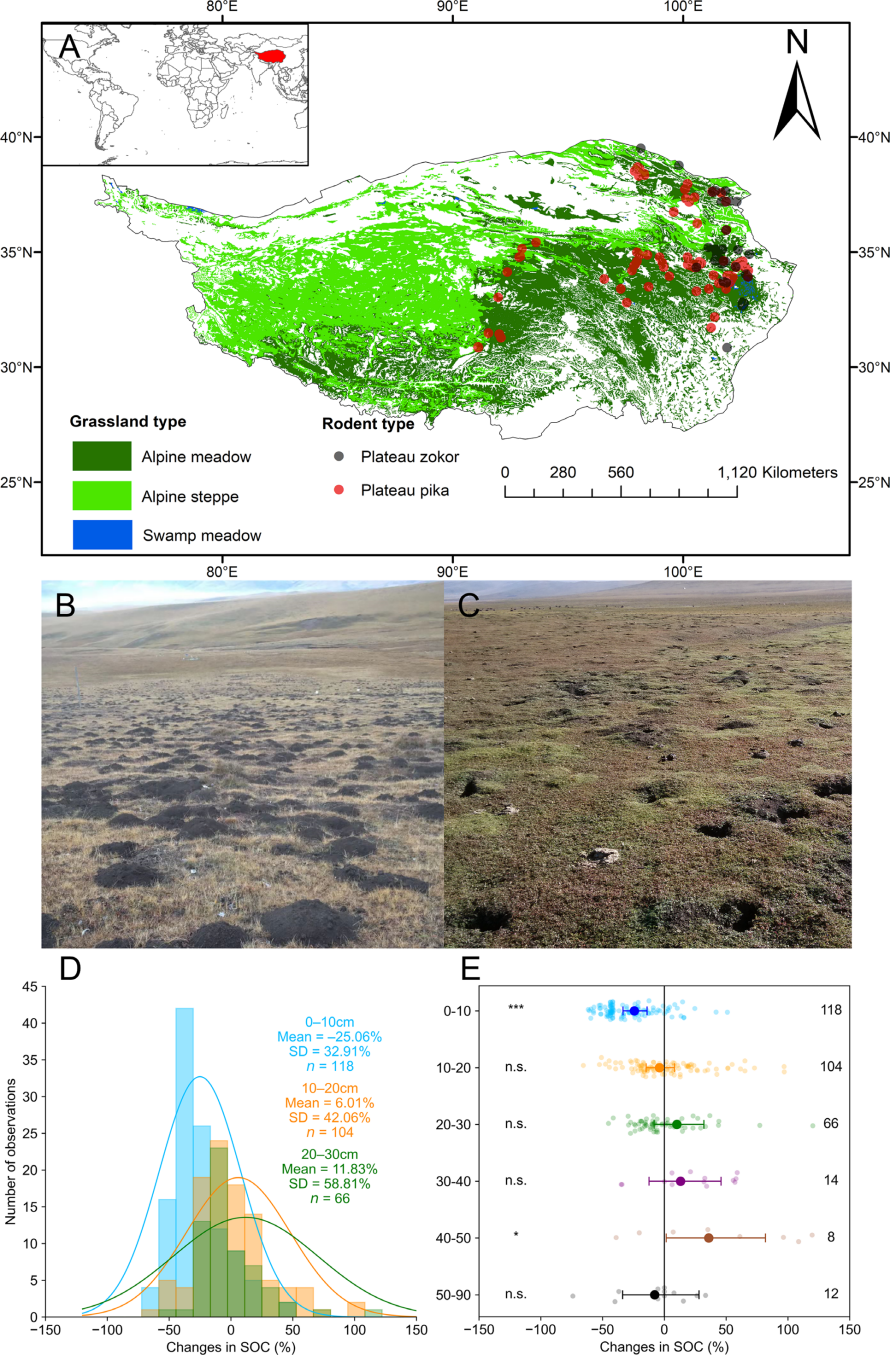
Changes in the Tibetan grassland SOC content due to rodent bioturbation. (A) Location of observation sites. Grassland type data are from ref. (45). (B) Landscapes of alpine meadows affected by Plateau zokor bioturbation. (C) Landscapes of alpine steppes affected by Plateau pika bioturbation. Photos B and C by L. Ma. (D) Distribution of changes in SOC contents in the 0 to 10, 10 to 20, and 20 to 30 cm soil layers (data for the soil layers 30 to 40, 40 to 50, and 50 to 90 cm are not shown because fewer observations were available). (E) Changes in the SOC contents of different soil layers (0 to 10, 10 to 20, 20 to 30, 30 to 40, 40 to 50, and 50 to 90 cm) due to rodent bioturbation. The larger solid circles and horizontal error bars denote the weighted means of changes and their 95% CIs. The smaller circles and numbers indicate individual changes due to rodent bioturbation and the number of observations, respectively. The asterisks *, **, and *** indicate significance at the levels of *P* < 0.05, 0.01, and 0.001, respectively, and n.s. indicates no significance (for details, see Meta-analysis in the “Methods” section).

The final database consisted of 322 paired SOC in-situ observations from 89 studies. Most measurements were from the eastern and northeastern Tibetan Plateau and some measurements were from the hinterlands of the Plateau, with measurements for alpine meadows representing 80% of all the data (Fig. 1A).

To systematically evaluate rodent bioturbation effects on SOC contents and stocks and to identify the underlying mechanisms, we extracted, in addition to SOC, information on other environmental variables (if available):

- Grassland characteristics: geographic location (latitude, longitude, altitude), time elapsed since the initial bioturbation, grassland type (steppe, meadow, swamp meadow), grassland background (grazing *vs*. ungrazed), burrow tunnel/mound density, and bioturbation affected grassland areas;
- Environmental conditions: mean annual temperature [MAT], mean annual precipitation [MAP], soil water content [SWC], soil temperature [*T*_s_], soil bulk density [BD], and soil pH [pH];
- Soil nutritional conditions: soil total nitrogen [STN], soil total phosphorus [STP], soil ammonium [${\mathrm{NH}}_{4}^{+}$], soil nitrate [${\mathrm{NO}}_{3}^{-}$], and soil dissolved organic carbon [DOC];
- Soil microbial parameters: microbial biomass carbon [MBC] and microbial biomass nitrogen [MBN];
- Vegetation: aboveground biomass [AGB], belowground biomass [BGB], biodiversity and species richness.

More information can be found in Supplementary data file_Meta-data.

### Meta-analysis of rodent bioturbation effect

To assess the rodent bioturbation effect sizes of SOC and environmental variables (e.g., BD, *T*_s_, etc.), the log-transformed response ratio (LnRR) was used (36):


(1) $$\mathrm{Ln}(\mathrm{RR})\;=\;\mathrm{Ln}\frac{X_{t}}{X_{c}}.$$

The results are presented as the relative % change (*e*^lnRR^–1) × 100). The variance (*v*) of RR was estimated using the following equation:


(2) $$v\;=\;\frac{{\mathrm{SD}}_{t}^{2}}{\;n_{t}\;X_{t}^{2}}\;+\;\frac{{\mathrm{SD}}_{c}^{2}}{\;n_{c}\;X_{c}^{2}},$$

where *X*_t_ and *X*_c_ indicate the means of the treatment (i.e., rodent bioturbated grassland) and control (i.e., undisturbed grassland), SD_t_ and SD_c_ indicate the SDs of the treatment and control and *n*_t_ and *n*_c_ indicate the numbers of replicates in the treatment and control, respectively.

However, the LnRR scalar cannot be applied to variables such as AGB, BGB, Shannon index, and plant species richness because approximately 76% of the studies reported the changes of these variables from positive values (in control) to zero values (in treatment) due to rodent bioturbation. Therefore, to calculate the effect sizes in these cases, we adapted the standardized mean difference metric “Hedges” *d*’ (*d*) to present the effect size (37), which was calculated as follows


(3) $$d=\left(1-\frac{3}{4\;(n_{t}+n_{c}-2)-1}\right)\;\frac{X_{t}-X_{c}}{S}.$$

The standard deviation (*S*) of *d* was calculated using the following equation:


(4) $$S=\sqrt{\frac{(n_{T}-1){\mathrm{SD}}_{T}^{2}+(n_{C}-1){\mathrm{SD}}_{C}^{2}}{n_{T}+n_{C}-2}}.$$

The *v* of *d* was estimated using the following equation:


(5) $$v\;=\;\frac{n_{t}\;+\;n_{c}}{n_{t}\;n_{c}}\;+\;\frac{d^{2}}{2\;(n_{t}\;+\;n_{c})},$$

where *X*_t_, *X*_c_, SD_t_, SD_c_, *n*_t_, and *n*_c_ have been described previously.

The weighted RR or *d* was calculated by individual RR or *d* with bias-corrected 95% CIs using the rma.mv function in the metafor package in R software (R core team, 2019) (38). This package allowed us to assign the variable “study” a random effect so that effects within the same study could be eliminated and the number of replicates could be considered as the weight (38). The impact of rodent bioturbation on a response variable was considered significant if the 95% CI did not overlap with 0 (38). Differences between subgroups were considered significant if the 95% CIs did not overlap with each other (38). The frequency distribution of RR for SOC was calculated to test variability among individual studies using the Gaussian function (i.e., normal distribution) (39).

### Estimation of the net changes in SOC stocks from Tibetan grasslands due to rodent bioturbation

Rodent bioturbation widely occurs in the Tibetan grasslands (40, 41, 42) and results in the mobilization of organic C in soil profiles. In this study, the net annual organic C loss or gain (Tg C yr^–1^) due to rodent bioturbation in the different soil layers (i.e., 0 to 10, 10 to 20, 20 to 30, 30 to 40, 40 to 50, and 50 to 90 cm) and the overall profile (i.e., 0 to 90 cm) were estimated for the alpine meadows and steppes, respectively. For this purpose, we first determined the net annual change rate of SOC content (△SOCC, g C kg^–1^ dry soil yr^–1^) due to rodent bioturbation using the following equation:


(6) $$△\mathrm{SOC}C_{i}\;=\frac{\mathrm{SO}C_{t,\;i}\;-\;\mathrm{SO}C_{c,\;i}}{\mathrm{Duration}}\;,$$

where SOC_t_ and SOC_c_ represent the SOC content (g C kg^–1^ dry soil) in soil layer *i* (i.e., *i* = 0 to 10, 10 to 20, 20 to 30, 30 to 40, 40 to 50, and 50 to 90 cm) and Duration indicates the years elapsed since the initial rodent bioturbation.

Furthermore, the net annual change rate of SOC stock (△SOCS, Tg C yr^–1^) due to rodent bioturbation was calculated as follows


(7) $$△\mathrm{SOC}S_{i,j}\;=\;\sum A_{j}\;\times \;△\mathrm{SOC}C_{i,j}\;\times \;BD_{i,j}\;\times \;H_{i}\times \frac{(1\;-\;C_{i,j})}{100},$$

where A*_j_* is the bioturbated area (10^3^ km^2^) in grassland type *j* (i.e., *j* = meadow or steppe), △SOCC*_i, j_* is the net annual change rate of SOC content (g C kg^–1^ yr^–1^), BD*_i, j_* is the mean bulk density (g cm^–3^) in the nonbioturbated grasslands, *H* is the soil layer thickness (cm), and *C_i, j_* is the volume percentage of the fraction >2 mm in layer *i* (i.e., *i* = 0 to 10, 10 to 20, and 20 to 30 cm) in grassland type *j* (i.e., *j* = meadow or steppe), respectively. We noted that the BD in the nonbioturbated grasslands was used for calculations because our meta-results showed that rodent bioturbation did not significantly influence BD across different soil layers (Fig. S2B). The same method was used for estimating net changes in SOC stocks due to climate change or land-use change (43).

### Data analysis

We classified all the collected data into soil layers of 0 to 10, 10 to 20, 20 to 30, 30 to 40, 40 to 50, and 50 to 90 cm in this study. Since not all data were within the 10-cm interval for each soil layer, we reclassified those soil layers on the basis of the median sampling soil depth (44). Specifically, we reclassified the soil layers 0 to 5 cm into 0 to 10, 0 to 20 and 10 to 20, 20 to 40 and 30 to 40 cm, etc.

Normality tests showed that the distribution of values for relative changes (i.e., %) in individual SOC content was either negatively (0 to 10 cm) or positively (>30 cm) skewed (Fig. 1D). The meta-analysis for SOC and environmental variables was performed using the metafor package in R software (R core team, 2019). The dependence of changes in SOC content on environmental variables such as MAT, MAP, STN, BD, etc., was evaluated using a linear (lm () function within R) or nonlinear (nls () function within R) regression analysis. Spearman’s correlation was performed between the changes in SOC content and environmental and biological variables using the “corrplot” and “Hmisc” packages within R software.

To estimate the uncertainty of the net annual changes in SOC stock of the Tibetan grasslands due to rodent bioturbation, net annual changes in SOC stock data were categorized by Tibetan grassland type (i.e., meadow and steppe) and soil layer (i.e., 0 to 10, 10 to 20, 20 to 30, 30 to 40, 40 to 50, and 50 to 90 cm). The software R i 386 3.6.1 (ggplot 2 package), ArcMap 10.7, and Python 3.9 were used for statistical analysis and graphics. The data are expressed as the mean values plus 95% CIs.

## Results

### Changes in the soil profile SOC content due to rodent bioturbation

Changes for the topsoil (0 to 10 cm) SOC content in the Tibetan grasslands due to rodent bioturbation were left skewed, while those in the deeper layers of soils (10 to 20 and 20 to 30 cm) were neutrally distributed (Fig. 1D). Across all studies, our meta-analysis showed that rodent bioturbation significantly decreased the entire soil profile (*P* < 0.05) and topsoil (*P* < 0.001) SOC contents (Figs. 1E and S1). However, the negative impacts of rodent bioturbation on SOC contents in the Tibetan grasslands were gradually decreased in the 10 to 20, 20 to 30, and 30 to 40 cm soil layers (although not significantly), while it showed significantly positive impacts in the 40 to 50 cm layer, i.e., it significantly (*P* < 0.05) increased SOC contents, and no significant impacts again in the 50 to 90 cm layer (Fig. 1E).

**Fig. 2.**
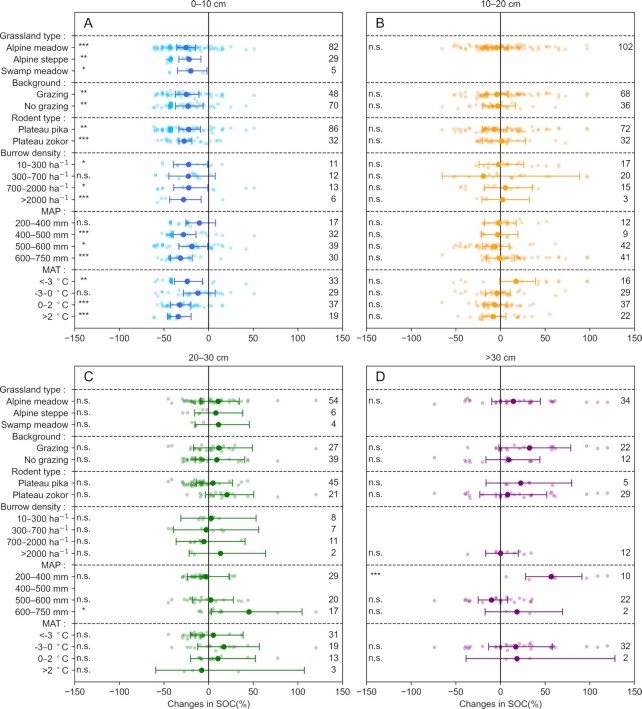
Changes in the Tibetan grassland SOC content due to rodent bioturbation under different conditions. (A) Changes in SOC in the 0 to 10 cm layer under different conditions. (B) Changes in SOC in the 10 to 20 cm layer under different conditions. (C) Changes in SOC in the 20 to 30 cm layer under different conditions. (D) Changes in SOC in the >30 cm layer under different conditions (note that observations for the 30 to 40, 40 to 50, and 50 to 90 cm soil layers were mixed to increase sample sizes, these are labeled >30 cm). The larger solid circles and horizontal error bars denote the weighted means of changes and their 95% CIs. The smaller circles and numbers indicate individual changes due to rodent bioturbation and the number of observations, respectively. The asterisks *, **, and *** indicate significance at the levels of *P* < 0.05, 0.01, and 0.001, respectively, and n.s. indicates no significance (for details, see Meta-analysis in the “Methods” section).

Subgroup analysis for SOC content (Fig. 2A to D) also supported the observation that rodent bioturbation significantly decreased the contents of SOC in the topsoil (0 to 10 cm) but showed no significant effects on the contents of SOC in the deeper layers of soils (note that observations for the 30 to 40, 40 to 50, and 50 to 90 cm soil layers were mixed to increase sample sizes, these are labeled >30 cm). However, the magnitudes of changes in the topsoil (0 to 10 cm) SOC contents due to rodent bioturbation varied to some extent among grassland types, grassland background, rodent type, etc. (Fig. 2A to D). Among all the grassland types, rodent bioturbation significantly decreased topsoil SOC. Relative to the background for grasslands, rodent bioturbation significantly reduced the topsoil SOC content in both grazing and nongrazing grasslands. With respect to rodent type, both Plateau pika and Plateau zokor resulted in significant SOC content losses. In terms of burrow density, decreases in SOC content exhibited a gradual increasing trend with increasing burrow densities from 10 to 300 ha^–1^ and >2,000 ha^–1^, due to rodent bioturbation (Fig. 2A).

Changes in the Tibetan grassland topsoil SOC contents due to rodent bioturbation depended on meteorological conditions, in addition to grassland and rodent properties (Fig. 2A). Decreases in the Tibetan grassland topsoil SOC contents gradually increased with increasing mean annual temperature (from <–3 to >2°C) and mean annual precipitation (from 200 to 400 and 650 to 750 mm). As shown in Fig. 2B to D, no significant effects of rodent bioturbation on SOC were observed in the deeper soil layers under different conditions.

### Changes in soil property and plant variables due to rodent bioturbation

**Fig. 3.**
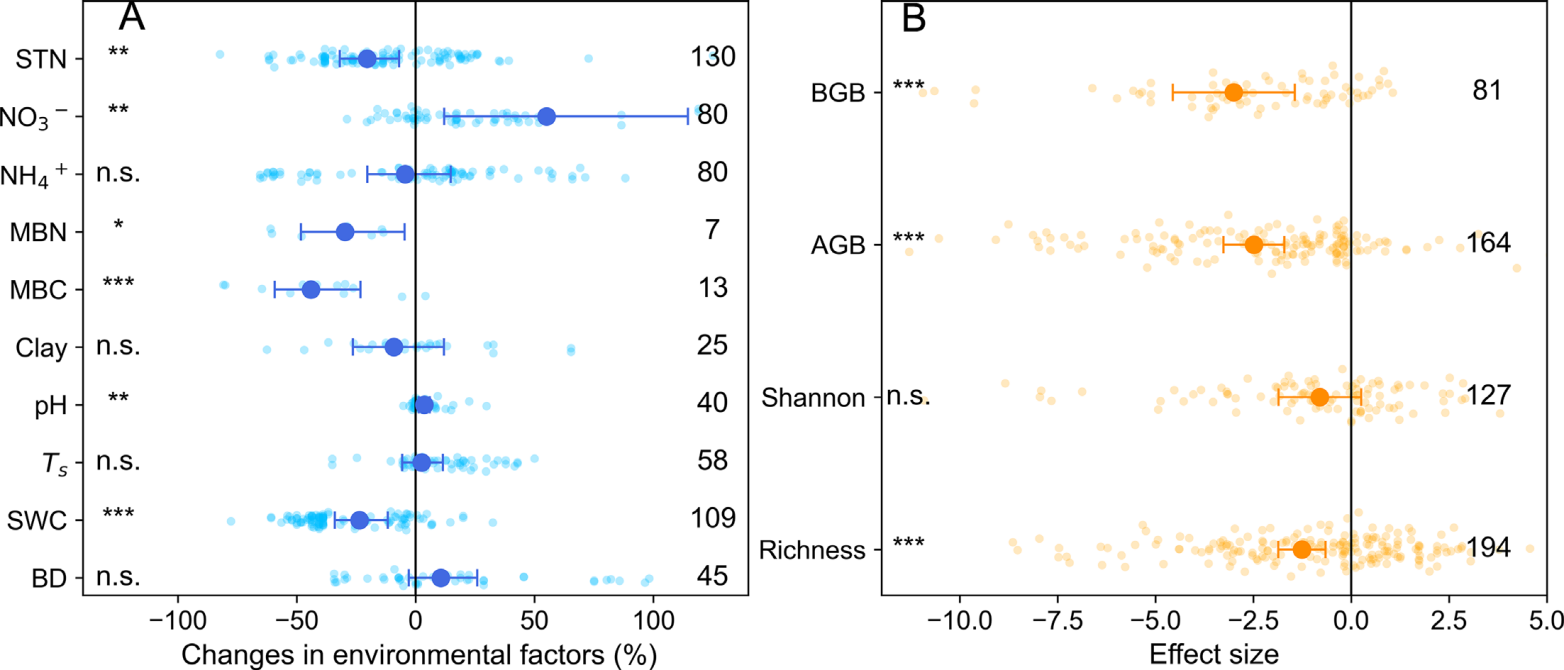
Changes in the Tibetan grassland environmental and biological variables due to rodent bioturbation. (A) Changes in environmental variables. (B) Effect size in biological variables (two different scalars were used for assessing the rodent bioturbation effects on environmental and biological variables, respectively, as the differences in the intrinsic attributes of values between the control and treatments for these two types variables, details see Meta-analysis in the “Methods” section). MBN, microbial biomass nitrogen; MBC, microbial biomass carbon; ST, soil temperature; SWC, soil water content; BGB, belowground biomass; AGB, aboveground biomass; Shannon, Shannon diversity index; Richness, plant species richness. Soil variables were only available for the topsoil (i.e., 0 to 10 cm). The larger solid circles and horizontal error bars in (A) denote the weighted means of changes and their 95% CIs. The larger solid circles and horizontal error bars in (B) denote the weighted Hedge’s *d* with 95% CIs. The smaller circles and numbers indicate individual changes due to rodent bioturbation and the number of observations, respectively. The asterisks *, **, and *** indicate significance at the levels of *P* < 0.05, 0.01, and 0.001, respectively, and n.s. indicates no significance (for details, see Meta-analysis in the “Methods” section).

On average, rodent bioturbation significantly decreased soil total nitrogen (STN) in the Tibetan grassland topsoil (0 to 10 cm) (Fig. 3A) but exerted no significant effects on that in the deeper soil layers (10 to 20, 20 to 30, 30 to 40, 40 to 50, and 50 to 90 cm) (Fig. S2A). Comparable trends were observed for the changes in STN and SOC contents for the Tibetan grasslands due to rodent bioturbation (Figs. 1E and S2A). Overall, rodent bioturbation shown nonsignificant effects on soil BD across all soil layers, but the increasing trend in BD decreased with increasing soil depth (Figs. 3A and S2B).

Since most soil variables were measured in the topsoil of the Tibetan grasslands. We mainly extracted these variables from the topsoil for meta-analysis. Overall, rodent bioturbation significantly increased soil ${\mathrm{NO}}_{3}^{-}$ contents and pH, while significantly decreasing MBN, MBC, and SWC (Fig. 3A). Rodent bioturbation also significantly reduced AGB, BGB, and species richness (in expressed as “Hedges” *d*’, see the “Methods” section) (Fig. 3B). However, rodent bioturbation showed nonsignificant effects on soil ${\mathrm{NH}}_{4}^{+}$ content, soil clay content, ST, and Shannon diversity (Figs. 3A and B).

### Drivers of the changes in topsoil SOC content due to rodent bioturbation

**Fig. 4.**
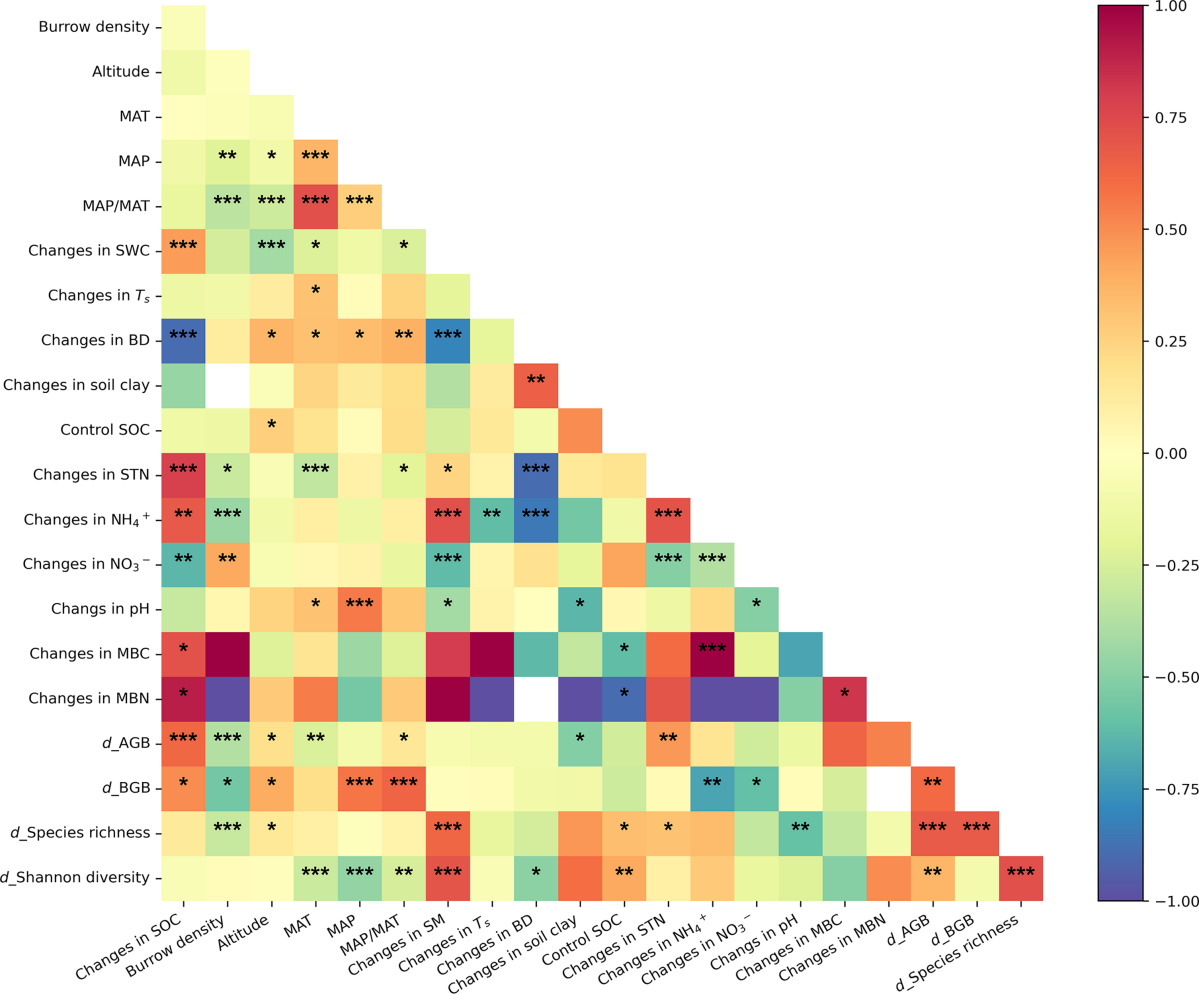
Spearman’s correlations between changes in topsoil (0 to 10 cm) SOC content and environmental and biological variables. *T*_s_, soil temperature. The asterisks *, **, and *** indicate significance at the levels of *P* < 0.05, 0.01, and 0.001, respectively, and n.s. indicates no significance.

Spearman’s correlation analysis showed that the changes in the topsoil (0 to 10 cm) SOC content due to rodent bioturbation were negatively correlated with the changes in ${\mathrm{NO}}_{3}^{-}$ contents, and BD in topsoil (0 to 10 cm) (Fig. 4); and they were positively correlated with the changes in STN contents, ${\mathrm{NH}}_{4}^{+}$ contents, MBC contents, MBN contents, and SWC in the topsoil, and with the changes in Hedge’s *d* of AGB and BGB (Fig. 4).

### Net changes in SOC stocks in the Tibetan grasslands due to rodent bioturbation

**Fig. 5.**
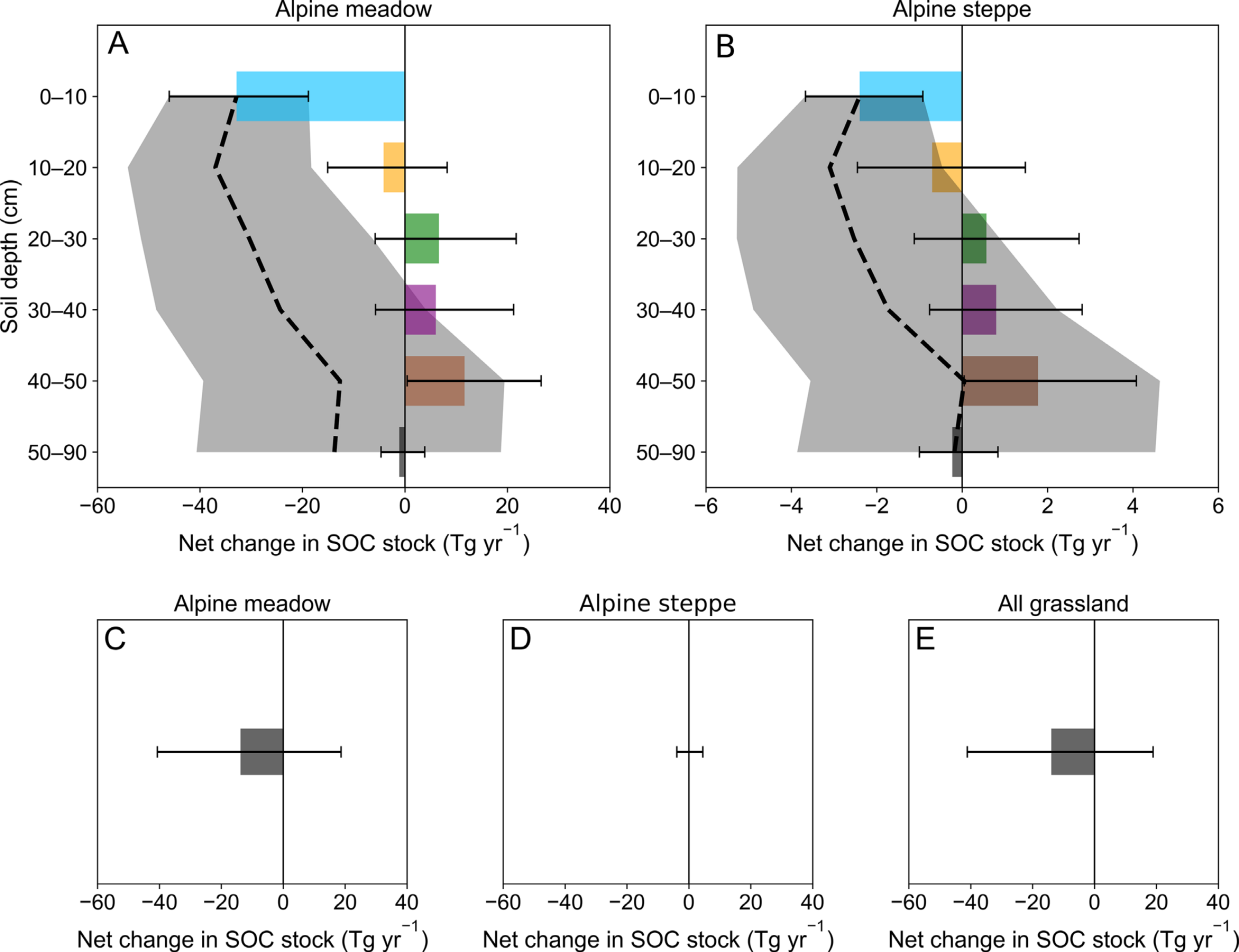
Net changes in the Tibetan grassland SOC stocks due to rodent bioturbation in each soil layer and across the overall soil profile. (A) Net changes and net cumulative changes in alpine meadow SOC stock due to rodent bioturbation in each soil layer (horizontal bars) and across the overall soil profile (dashed line). (B) Net changes and net cumulative changes in alpine steppe SOC stock due to rodent bioturbation in each soil layer (horizontal bars) and across the overall soil profile (dashed line). (C) Net changes in alpine meadow SOC stock due to rodent bioturbation across the overall soil profile (horizontal bar). (D) Net changes in alpine steppe SOC stock due to rodent bioturbation across the overall soil profile (horizontal bar). (E) Net changes in alpine grassland (meadow + steppe) SOC stock due to rodent bioturbation across the overall soil profile (horizontal bar). The horizontal error bars and shaded areas represent 95% CIs, with those covering the zero line indicating nonsignificant effects due to rodent bioturbation.

For alpine meadows, rodent bioturbation resulted in a significant net loss in the SOC stock of the topsoil (0 to 10 cm) of −32.8 Tg C yr^–1^, with a 95% CI of −46.0 to −18.8 Tg C yr^–1^, but showed nonsignificant effects on the SOC stocks in the 10 to 20, 20 to 30, 30 to 40, and 50 to 90 cm soil layers (Fig. 5A). Rodent bioturbation triggered a significant increase in the SOC stock of the 40 to 50 cm soil layer of +11.6 Tg C yr^–1^, with a 95% CI of +0.5 to +26.6 Tg C yr^–1^ (Fig. 5A). For alpine steppes, similar trends were observed, showing that rodent bioturbation resulted in a significant net loss in SOC stocks in the topsoil (0 to 10 cm) of −2.4 Tg C yr^–1^, with a 95% CI of −3.7 to −0.9 Tg C yr^–1^, while triggering a significant increase in the SOC stocks of the 40 to 50 cm soil layer of +1.8 Tg C yr^–1^, with a 95% CI of +0.1 to +4.1 Tg C yr^–1^ (Fig. 5B). Rodent bioturbation showed nonsignificant effects on SOC stocks in the 10 to 20, 20 to 30, 30 to 40, and 50 to 90 cm soil layers (Fig. 5B).

In the topsoil (0 to 10 cm) and the 0 to 30 cm soil layer, rodent bioturbation led to significant net losses of SOC of approximately −35.2 Tg C yr^–1^ (95% CI of −48.5 to −21.1 Tg C yr^–1^) and −32.9 Tg C yr^–1^ (95% CI of −54.2 to −8.6 Tg C yr^–1^), respectively, across the Tibetan grasslands (Fig. 5A and B). However, when the overall 0 to 90 cm soil layers were considered (by summing up net changes in the SOC stock for each soil layer), rodent bioturbation exerted nonsignificant net losses on SOC stock (–13.9 Tg C yr^–1^, with a 95% CI of −41.1 to +18.3 Tg C yr^–1^) across the Tibetan grasslands (Fig. 5C to E).

## Discussion

### Mechanisms that underly the changes in depth-dependent SOC content due to rodent bioturbation

Rodent bioturbation has been discussed for decades. Previous studies mainly focused on in-situ SOC observations in surface soil layers at the site-specific scale. The large spatial heterogeneities in soil and plant properties, as well as variations in rodent types and densities, etc., make the situ SOC observations varied from site to site. In this study, we first time used the meta-analysis and upscaling approaches to evaluate the rodent bioturbation impacts on SOC contents and stocks across the Tibetan grasslands, one of the representative grassland ecosystems across Eurasian grasslands.

**Fig. 6.**
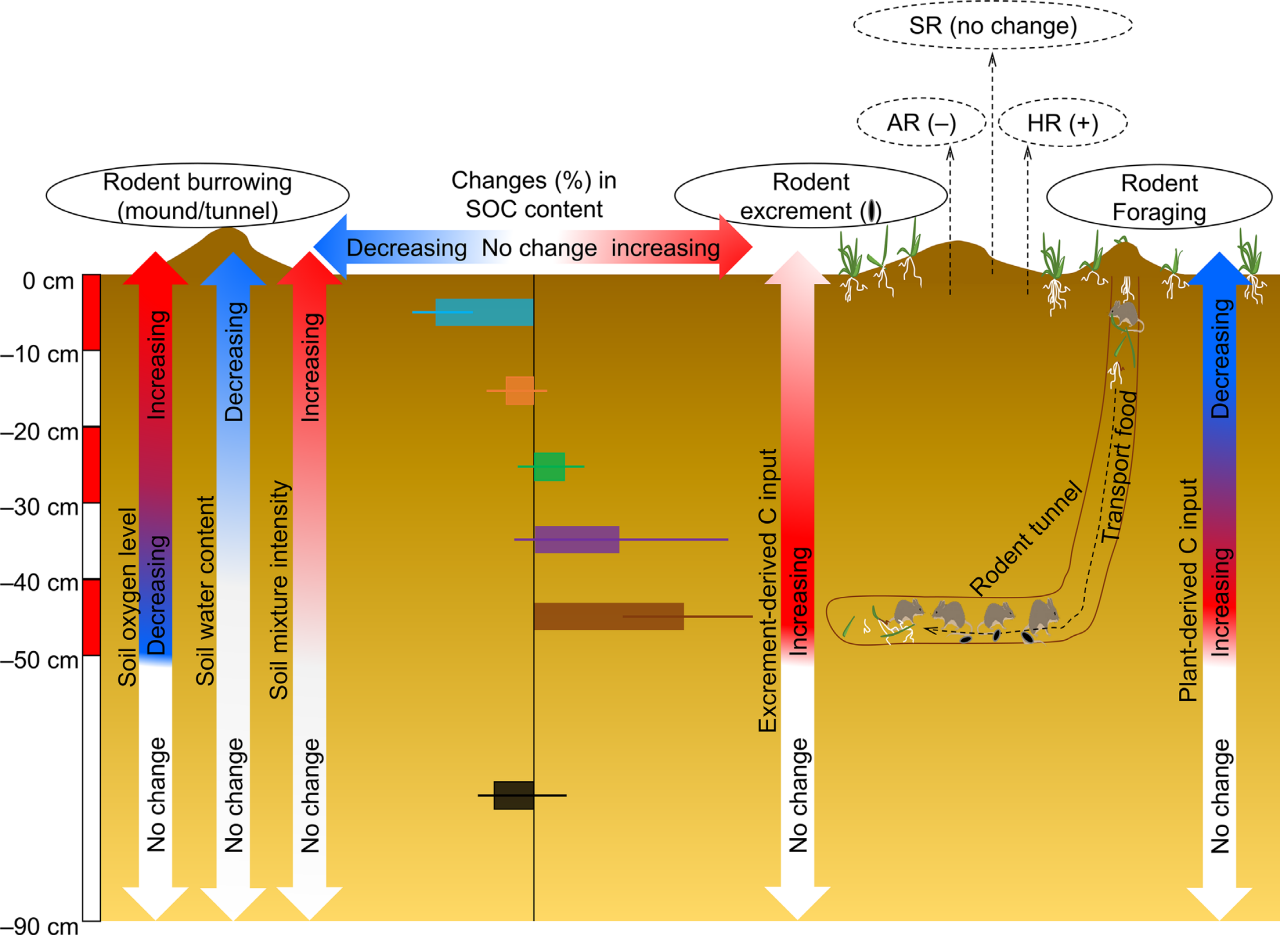
Conceptual model describing the potential mechanisms driving the changes in depth-dependent SOC content resulting from rodent bioturbation. This conceptual model was built based on the meta-results for the changes in SOC content and environmental and biological variables resulting from rodent bioturbation (for details, see Figs. 1E and 3A and B), as well as some deductions based on our current understanding of rodent bioturbation. These deductions should be tested in future studies. SR = HR + AR (54, 55). “(+),” increase resulting from rodent bioturbation; “(**–**),” decrease resulting from rodent bioturbation; “no change,” no change resulting from rodent bioturbation.

Our results demonstrated that the impacts of rodent bioturbation on the Tibetan grassland SOC contents were depth-dependent (Figs. 1E and 2A to D). The depth-dependent phenomena have been reported in managed agricultural soils worldwide (46–48). The significant reduction in SOC content in the topsoil (0 to 10 cm) due to rodent bioturbation was collectively caused by the following reasons. First, rodent burrowing activities loosened topsoil during the early stages of bioturbation and significantly reduced soil water content (Fig. 3A), and consequently, topsoil organic matter decomposition was enhanced in the improved aeration area of the topsoil [Fig. 6 and refs. (31–33)]. This was further supported by the observation that net decreases in SOC content were significantly correlated with net decreases in soil water content across the studied sites (Fig. 4). This explanation was also indirectly evidenced by the significant increases in soil ${\mathrm{NO}}_{3}^{-}$ contents due to rodent bioturbation, as increases in soil ${\mathrm{NO}}_{3}^{-}$ contents were mainly derived from the organic matter nitrification process under aerated conditions (49). Although rodent bioturbation loosened the topsoil and thus was assumed to lower soil BD, the significant reductions in BGB in the topsoil due to rodent bioturbation implied that the volume of space occupied by roots was replaced by solid soil and consequently BD increased compared with nonbioturbated grassland soils (40). Furthermore, the net loss in topsoil SOC due to rodent bioturbation gradually compacts the soil, consequently increasing BD (50). The observed negative exponential trend between SOC content and BD further supports this explanation (51). In addition, the rodent-made mounds gradually flattened over time due to gravity and consequently compact the soil (52). These factors together explain the nonsignificant changes and even slight increases in topsoil BD due to rodent bioturbation. Second, rodent foraging significantly reduced AGB and BGB and consequently largely reduced plant-derived C inputs to the topsoil [Fig. 6 and ref. (53)]. This explanation was also supported by the observations that decreases in topsoil SOC contents were closely related to decreases in AGB and BGB across the studied sites (Fig. 4). The significant decreases in BGB could significantly inhibit soil autotrophic respiration (AR), as AR is primarily derived from belowground roots (54). In contrast, we observed that soil respiration (SR) was not significantly affected by rodent bioturbation (Fig. S3A). We therefore deduced that soil heterotrophic respiration (HR) may be significantly enhanced due to rodent bioturbation, as SR includes HR and AR (54, 55). This also partly explained the first possible reason for the enhancement of topsoil organic matter decomposition under the improved aeration areas of topsoil. Third, burrowing activities can transfer several tons of soil from the deeper layers to the ground surface and a dilution effect is expected to occur when the soils are sufficiently mixed (56). In this study, we were unable to separate the contributions of each to the observed declines in topsoil SOC contents due to rodent bioturbation; therefore, more research is required to fill this knowledge gap in future studies.

The significant increases in SOC contents at the 40 to 50 cm depth due to rodent bioturbation were jointly contributed by: (1) the additional plant-derived C inputs from the storage of grasses and seeds in rodent nests (Fig. 6); (2) increases in SOC content due to the deposition of feces and urine in the rodent nests (Fig. 6); and (3) potential increases in SOC content due to the mixing of the upper and deeper layers of soil (Fig. 6). Additionally, rodent respiration resulted in more anaerobic soil conditions relative to nonbioturbated sites (Fig. 6), which resulted in a relative inhibition of the decomposition of SOC in the deeper layers of soil. The nonsignificant impacts on the SOC contents at the 50 to 90 cm depth indicated that rodent bioturbation, to a large extent, was limited a maximum depth of 50 cm below the ground surface (50). Similarly, we were unable to separate the contributions of each to the observed rodent bioturbation-induced enhancements in the 40 to 50 cm SOC contents, and this also warrants more research in future studies. Our study also showed that the changes in SOC content, regardless of soil depth, were not significantly different among the different grassland types, grassland backgrounds, rodent types. However, we observed that the decreases in topsoil SOC content increased with increasing burrowing densities from <300 to >2,000 ha^–1^ (Fig. 2A), suggesting that rodent bioturbation impacts on SOC contents of the Tibetan grasslands were independent of grassland type, land-use background, and rodent type, but was greatly affected by the rodent burrowing density (22). This result indicates that rodent burrowing activities should be controlled within a certain range, which could effectively reduce the losses of SOC in the Tibetan grasslands.

### Net changes in SOC stocks in the Tibetan grasslands due to rodent bioturbation

The depth-dependent responses in the Tibetan grassland SOC content resulting from rodent bioturbation highlighted the importance of including entire soil profile to achieve robust quantifications of annual net losses in SOC stocks due to rodent bioturbation. Consistent with previous studies (22, 57), net SOC loss occurred in the 0 to 30 cm layer due to rodent bioturbation. These net losses could completely offset the net annual SOC (0 to 30 cm) accumulation of 32.0 Tg C yr^–1^ (95% CI: 17.4 to 46.7 Tg C yr^–1^) in the Tibetan grasslands from 2000 to 2010 (7). However, by considering the entire soil profile affected by rodent bioturbation (i.e., 0 to 90 cm), it was seen that rodent bioturbation caused nonsignificant net losses in SOC stocks in the 0 to 90 cm layer across the Tibetan grasslands (Fig. 5C to E). This is mainly due to that SOC losses in the surface layer being offset by the SOC increases in the 40 to 50 cm soil layer (Fig. 5A and B). These results collectively demonstrated that all the soil layers must be considered to robustly quantify the net changes in SOC stocks due to anthropogenic activities or climate change.

Although rodent bioturbation showed a nonsignificant effect on net changes in overall soil profile SOC stocks across the Tibetan grasslands, significantly decreases in AGB and BGB due to rodent bioturbation (Fig. 3B) will inevitably reduce net C uptake in the alpine grasslands and thus enhance climate warming. Moreover, the significant decrease in plant species richness due to rodent bioturbation (Fig. 3B) threatens plant biodiversity in the alpine grasslands. From these perspectives, alpine grasslands should be prioritized in conservation efforts.

### Limitations and the way forward

Our study was conducted in the Tibetan grasslands, a regional-scale study; however, these grasslands have large SOC stocks and is sensitivity to climate change (e.g., warming) and disturbances (rodent activity). Therefore, conducting such studies in the Tibetan grasslands have great implications for the SOC dynamics in global grassland ecosystems. In addition, most measurements were conducted in alpine meadows and steppes while only five observations were performed in alpine swamp meadows, therefore, the meta-analysis results for alpine swamp meadows are still questionable. Overall, alpine swamp meadows exhibited higher SOC and soil water contents than other types of Tibetan grasslands (9). Thus, we postulate that larger decreases in SOC stock will occur if they are bioturbated by rodents. Over the past decades, a large number of swamp meadows in the eastern Tibetan Plateau have experienced severe degradation due to rodent bioturbation (58, 59). We therefore propose initiating more measurements in the alpine swamp meadows, although the areas occupied by alpine swamp meadows are much smaller than those of alpine meadows and steppes (7). Moreover, although we found that the responses of SOC content to rodent bioturbation were depth-dependent, the relatively smaller sample sizes of the deeper soil layers (i.e., >30 cm) might have introduced some uncertainties to our results. Conducting more targeted measurements would be beneficial for better constraining the depth-dependent phenomenon. In addition, rodent bioturbation may also significantly influence the soil–atmosphere exchanges of the greenhouse gases methane (CH_4_) and nitrous oxide (N_2_O), which have global warming potentials of that are 27- and 273-fold greater than CO_2_ over a 100-year timescale (60). Therefore, a full assessment of the net changes in SOC stocks and CH_4_ and N_2_O fluxes due to rodent bioturbation would be beneficial for an in-depth understanding of their roles in climate change.

In summary, our meta-analysis revealed that rodent bioturbation impacts on the SOC contents in the Tibetan grasslands were depth-dependent. A significant decrease in the topsoil SOC content was ascribed to the collective effects of enhanced organic matter decomposition under improved aeration conditions, reduced plant-derived C inputs due to rodent foraging activities, and dilution effects resulting from the mixing of the deeper and upper layers of soils. Significant increases in the deeper layer of soil (40 to 50 cm) SOC content occurred due to joint contributions from the increased C inputs from the storage of plant and seed food resources, and from the deposited feces and urine in the nests, as well as enrichment effects from the mixing of the upper and deeper layers of soils. Although our meta-results shown no significant SOC losses across entire disturbed soil profile, rodent bioturbation greatly decreases above- and belowground biomass as well as lowers species richness, and thus reduces net C uptake during subsequent growing seasons in alpine grasslands and further exacerbates climate warming. Therefore, the conservation of alpine grasslands can help maintain productivity and biodiversity and mitigate climate change.

## Data Availability

All data used in this study were submitted as Supplementary data files.
